# The associations of multimorbidity with fall- and fracture-related hospitalisations: the Busselton Healthy Ageing Study

**DOI:** 10.1007/s11657-025-01600-w

**Published:** 2025-08-29

**Authors:** Mark A Hoey, Kun Zhu, Kevin Murray, Chrianna Bharat, Robert H Eikelboom, Michael Hunter

**Affiliations:** 1 WA Country Health Service, Perth, Australia; 2 Medical School, The University of Western Australia, Perth, Australia; 3 Department of Endocrinology and Diabetes, Sir Charles Gairdner Hospital, Perth, Australia; 4 School of Population and Global Health, The University of Western Australia, Perth, Australia; 5 National Drug and Alcohol Research Centre, University of New South Wales, Sydney, Australia; 6 Centre for Ear Science, Medical School, The University of Western Australia, Perth, Australia; 7 Ear Science Institute Australia, Perth, Australia; 8 Department of Speech Language Pathology and Audiology, University of Pretoria, Pretoria, South Africa; 9 Busselton Population Medical Research Institute, Busselton, Australia

**Keywords:** Multimorbidity, Falls, Fractures, Multimorbidity patterns, Middle-aged adults, Cohort study

## Abstract

**Summary:**

In middle-aged adults, we evaluated the associations between multimorbidity count and patterns with fall- and fracture-related hospitalisations. Falls risk increased linearly with multimorbidity count, and certain multimorbidity patterns were associated with increased risks of falls and fractures. Multimorbidity count and pattern should therefore be considered when risk stratifying patients.

**Purpose:**

Although multimorbidity is recognised as a risk factor for falls and fractures, most studies are retrospective, and few have explored these relationships through statistically derived multimorbidity patterns. Our prospective cohort study with 4991 participants of the Busselton Healthy Ageing Study aged 45–69 years evaluated the associations of multimorbidity count and classes with incident fall- and fracture-related hospitalisations.

**Methods:**

Twenty-one morbidities were assessed at baseline, and four multimorbidity classes were identified using latent class analysis. Fall- and fracture-related hospitalisations were captured through the Western Australian Data Linkage System over a median follow-up of 7.9 years. Associations were examined using Cox regression models adjusting for sex, baseline age, lifestyle factors, and prior falls/fractures.

**Results:**

During follow-up, incident fall- and fracture-related hospitalisations were recorded for 177 (3.5%) and 197 (3.9%) participants, respectively. Each one-unit increase in multimorbidity count was associated with a 16% (95% CI, 7.8–25%) increased risk of fall-related hospitalisations. Multimorbidity scores of 9 and above (HR 2.32 [1.22–4.42]) showed an increased risk of fractures. Compared with the relatively healthy class, the cardiometabolic or mental health and musculoskeletal classes were associated with an increased risk of fall-related hospitalisations (HR 2.84 [1.76–4.59] and 1.78 [1.23–2.59], respectively). The cardiometabolic class was associated with an increased risk of fracture-related hospitalisations (HR 1.79 [1.04–3.07]).

**Conclusion:**

In middle-aged adults, we showed that multimorbidity count and certain multimorbidity patterns were associated with increased risk for fall- and fracture-related hospitalisations. Multimorbidity should therefore be considered when assessing a patient’s risk of falls and fractures.

## Introduction

With the ageing population, fall- and fracture-related hospitalisations present an increasing burden to the healthcare system. A fall is defined as an event which results in a person coming to rest inadvertently on the ground, floor, or other lower level. The World Health Organization estimates that over 37 million falls each year are severe enough to require medical attention, resulting in an annual loss of over 38 million disability-adjusted life years [1]. Falls are associated with increased disability and mortality and have been shown to lead to functional decline and significant healthcare costs [2, 3]. A range of risk factors for falls have been identified, including sociodemographic and psychological factors, chronic medical conditions, and poor physical functioning [4–6].

Fragility fractures are commonly defined as fractures resulting from low-energy trauma, such as a fall from a standing height or less, and occur most commonly at the hip, spine, and forearm [7]. An association between falls and an increased risk of fractures has been shown in several studies [8, 9], with at least 90% of forearm and hip fractures resulting from a fall to the ground [7]. However, only 5% of falls lead to a fracture [10]. Many studies have shown that low bone mineral density is associated with an increased risk of fractures [11, 12], with the addition of several clinical risk factors shown to increase predictive value [13]. Fragility fractures have been shown to incur a huge cost to both an individual’s quality of life and to overall health systems. In Australia, fragility fractures were estimated to result in a loss of 18% of quality-adjusted life years across all fractures over 12 months [14]. Identifying individuals at high risk of falls and fragility fractures is therefore important to facilitate targeted prevention strategies.

Multimorbidity, commonly referred to as the coexistence of two or more chronic medical conditions in an individual, is common [15]. In Australia and other developed countries, ageing populations have led to an increasing prevalence of multimorbidity. The Busselton Healthy Ageing Study (BHAS), a community survey of adults aged 45–69 years in Busselton, Western Australia, found 73% of the cohort had multimorbidity [16]. This value is consistent with similar studies in other countries [17, 18]. Research into the associations between multimorbidity, falls, and fractures has been limited. Most studies have been retrospective or cross-sectional [19–25], making it difficult to determine the directionality of the relationship. A small number of longitudinal studies have also been published, all finding multimorbidity to be a significant risk factor for falls, with the risk increasing with the number of chronic medical conditions [17, 18, 26].

Although measuring multimorbidity using overall disease counts can help identify individuals with complex healthcare requirements, it is unable to differentiate between an individual’s different types of diseases. The use of multimorbidity patterns or classes therefore has advantages. Multimorbidity patterns are statistically derived combinations of diseases that are aggregated based on the associations between them [27]. Very few studies have derived different multimorbidity patterns and explored their association with falls and fractures [17, 28, 29], necessitating further research in this area. A recent nationwide cohort study in Denmark found that multimorbidity clusters or patterns had a higher discriminative ability to assess fracture risk than simple individual morbidity counts [29].

In the BHAS, we characterised both the level and type of multimorbidity through overall multimorbidity count and replicable multimorbidity classes. In addition, potential bias has been reduced by using objective data in diagnosis where possible. Using detailed measures of multimorbidity collected in the BHAS linked with hospital admissions data, we investigated multimorbidity count and multimorbidity patterns as predictors of incident fall- and fracture-related hospitalisations over a median follow-up of 7.9 years in 4991 participants.

## Methods

### Study design and participants

The study participants were sourced from the BHAS, as detailed previously [30]. In summary, the BHAS is an observational community-based longitudinal cohort study of middle-aged adults (born 1946–1964) living in the City of Busselton, Western Australia. In Phase 1 (2010–2015), a comprehensive questionnaire, biospecimens, and clinical examination were used to collect baseline health data for 5107 participants. A multimorbidity analysis was completed for the 4991 participants with complete morbidity data who consented to data linkage and were included in the present study, resulting in four distinct patterns of multimorbidity [16]. Data was collected and stored for research purposes with informed consent from all participants and with ethics approval from the University of Western Australia (UWA HREC – 2021/ET00260).

### Outcomes—fall- and fracture-related hospitalisations

Fall- and fracture-related hospitalisations were captured through the Western Australia Data Linkage System, retrieved from the Western Australia Hospital Morbidity Data Collection, and defined using the International Statistical Classification of Diseases and Related Health Problems, 10th Revision [31]. Incident fall- and fracture-related hospitalisations were included from the date of the participant’s baseline visit until the first of the following outcomes: admission date for relevant hospitalisation, death, or the end of the follow-up period (31st December 2020).

Primary and secondary diagnostic codes recorded during presentation were included in defining the outcomes of interest. Fall-related hospitalisations were identified using ICD-10 codes W01, W05, W06, W07, W08, W10, W18, and W19 [31]. Fracture-related hospitalisations were identified using ICD-10 codes S02, S12, S22, S32, S42, S52, S62, S72, S82, S92, M80, T02, T08, T10, T12, and T14.2 [31]. Fractures of the face (S02.2 to S02.6), fingers (S62.5 to S62.7), and toes (S92.4 to S92.5) and fractures caused by motor vehicle injuries (V00 to V99) were excluded.

### Exposures—baseline morbidities and multimorbidity classes

A combination of self-reported doctor diagnosis, objective measures, and validated instrument scale scores was used to define 21 different morbidities at baseline, as previously detailed in the literature [16, 30]. The 21 different morbidities included in the analyses were all prevalent and chronic, predominantly diseases or symptoms, and chosen by BHAS multidisciplinary consensus [16]. Eight were defined by self-reported doctor diagnosis (arthritis, bowel disease, eye condition, asthma, thyroid disease, skin cancer, cancer (non-skin), stroke). Seven conditions were defined using objective measures, a validated instrument, or self-reported doctor diagnosis (liver disease, heart disease, obstructive sleep apnoea, diabetes, COPD, kidney disease, osteoporosis), and six were defined using only objective measures or a validated instrument (atopy, peripheral artery disease, hearing loss, depression/anxiety, bronchitis, low back pain). The prevalence of these conditions varied from 1.9 to 54.4%. Bronchitis and COPD were included in the analyses as two separate morbidities because, in contrast to the objective spirometry results used for defining COPD, bronchitis was defined based on self-reported symptoms. In addition, the two morbidities had differing prevalences in the cohort. The multimorbidity count was determined by the sum of these individual conditions.

The multimorbidity classes were determined using latent class analysis (LCA). LCA is a model-based statistical technique that has previously been used in separating a population into distinct classes and identifying patterns of multimorbidity [27], with this particular analysis previously detailed in the literature [16, 32]. In this LCA analysis, the four-class model was interpreted to be optimal, with the overall morbidity profiles of each class used for the naming of the classes: Class 1 — relatively healthy, Class 2 — predominantly respiratory and atopy, Class 3 — predominantly mental health and musculoskeletal, and Class 4 — predominantly cardiometabolic. The titles of the four multimorbidity classes were modified from the original LCA study [16] in order to better facilitate interpretation and to better match replicable profiles of multimorbidity identified in a recent systematic review [27]. Although Class 3 is heterogenous and includes a range of conditions, the most prevalent and clinically distinguishing were arthritis, depression/anxiety, and low back pain, supporting its title of predominantly mental health and musculoskeletal.

### Covariates

Covariates were measured at the baseline visit of the BHAS (Phase 1, 2010–2015). Demographic variables utilised in the multivariate adjusted models included age, sex, body mass index (BMI), tobacco smoking (never, former, current < 15/day, current = > 15/day), moderate/vigorous physical activity (median hours/week) [33], alcohol consumption (median glasses/week), and previous history of the outcome of interest (fall- or fracture-related hospitalisations). A previous history of the outcome of interest was determined according to whether there were any relevant hospitalisations in the 5 years prior to their BHAS Phase 1 baseline visit, identified using ICD-10 codes as stated for incident fall- and fracture-related hospitalisations.

### Statistical analysis

The mean and standard deviation (SD), or median and interquartile range (IQR), are included for continuous variables, and frequencies and percentages are included for categorical variables. Kaplan–Meier survival analyses were used to display the univariate relationship between measures of interest (multimorbidity LCA class) with fall- and fracture-related hospitalisations.

Using Cox proportional hazards regression, the association between measures of interest (multimorbidity count (continuous measure), multimorbidity LCA class) with fall- and fracture-related hospitalisations was examined using three models: (i) Model A, unadjusted; (ii) Model B, adjusted for age, BMI and sex; and (iii) Model C, Model B plus additionally adjusting for smoking status, physical activity (hours of moderate/vigorous exercise), number of alcohol glasses per week, and history of the same event in the 5 years prior to baseline (yes/no). The assumptions for Cox proportional hazards models were examined based on Schoenfeld residuals. Restricted cubic splines were used to investigate nonlinear relations of multimorbidity count and the outcome of interest. Linearity was assessed using a likelihood ratio test comparing nested models with and without spline terms. All results are presented as point estimates and 95% confidence intervals. The statistical analyses were performed using SAS Version 9.4 and R version 4.3.1 (R Foundation for Statistical Computing, Vienna, Austria).

## Results

Table 1 describes the cohort’s baseline demographics. Of the 4991 included in these analyses, 54.7% were female, and the majority had either never smoked (46.9%) or were ex-smokers (43.2%). The mean (SD) age of participants at baseline was 58.0 (5.8) years with a range of 45–69 years. Participants engaged in a median (IQR) of 4.5 [0, 14.0] h of moderate to vigorous physical activity and consumed a median (IQR) of 7.0 [1.4, 16.5] glasses of alcohol per week.

**Table 1:** Baseline characteristics of the study population

	**All participants** ***N* = 4991**
Age, y, mean (SD)	58.0 (5.8)
Sex, *n* (%)	
Female	2730 (54.7)
Male	2261 (45.3)
BMI, median (IQR)	27.5 [24.8, 30.9]
Moderate/vigorous physical activity (hrs/week), median (IQR)	4.5 [0.0, 14.0]
Alcohol consumption (glasses/week), median (IQR)	7.0 [1.4, 16.5]
Tobacco smoking, *n* (%)	
≥ 15 cigarettes a day	272 (5.4)
< 15 cigarettes a day	225 (4.5)
Ex-smoker	2154 (43.2)
Never	2340 (46.9)
Multimorbidity count (21 conditions), mean (SD)	2.7 (1.8)
LCA multimorbidity class, *n* (%)	
1 — relatively healthy	3517 (70.5)
2 — predominantly respiratory and atopy	525 (10.5)
3 — predominantly mental health and musculoskeletal	682 (13.7)
4 — predominantly cardiometabolic	267 (5.3)
Past 5-year fall-related hospitalisation, *n* (%)	
No	4931 (98.8)
Yes	60 (1.2)
Past 5-year fracture-related hospitalisation, *n* (%)	
No	4913 (98.4)
Yes	78 (1.6)

*BMI* body mass index, *LCA* latent class analysis

The prevalence of fall- and fracture-related hospitalisations 5 years prior to baseline was 1.2% and 1.6%, respectively. The mean number of chronic conditions at baseline was 2.7 (1.8). During the median (IQR) follow-up period of 7.9 [6.7, 9.2] years, fall- and fracture-related hospitalisations were recorded among 177 (3.5%) and 197 (3.9%) of participants, respectively. One hundred of the 177 (56.5%) participants with a fall-related hospitalisation also had a fracture-related hospitalisation, while 100 of the 197 (50.8%) fracture-related hospitalisations also had a fall-related hospitalisation. Table 2 describes the multimorbidity characteristics and number of events for the four multimorbidity classes.

**Table 2:** LCA multimorbidity class morbidity characteristics and number of outcome events

	**All**	**Class 1**	**Class 2**	**Class 3**	**Class 4**
	*N* = 4991	*N* = 3517	*N* = 525	*N* = 682	*N* = 267
Mean number of conditions	2.75	2.0	3.6	4.8	6.3
Condition, % prevalence					
Atopy	54.4	52.3	**81.9**	40.6	62.5
Arthritis	25.1	13.3	24.8	**77.1**	47.9
Bowel disease	23.3	16.6	22.1	**54.0**	35.2
Liver disease	23.1	21.9	23.2	16.0	**57.3**
Depression/anxiety	20.5	13.4	14.3	**54.8**	38.6
Bronchitis	17.7	11.9	20.4	**37.8**	37.1
Eye condition	14.6	10.8	12.0	**30.4**	29.2
Asthma	14.2	0.1	**99.0**	14.4	32.6
Heart disease	9.7	7.7	9.1	7.2	**44.2**
Thyroid disease	8.6	5.1	7.6	**25.8**	12.4
Low back pain	8.5	1.6	4.8	**37.4**	32.6
Skin cancer	8.1	8.7	4.8	**8.7**	6.4
OSA	8.0	5.8	2.5	12.3	**37.1**
Hearing loss	7.1	6.0	3.6	11.6	**16.1**
Diabetes	7.0	4.2	0	4.3	**64.8**
Non-skin cancer	6.7	5.0	6.3	**14.2**	10.9
COPD	5.7	2.7	**19.2**	8.1	12.7
Kidney disease	5.2	3.4	2.7	10.1	**21.0**
Osteoporosis	3.4	2.7	4.2	**7.6**	0.4
Stroke	2.1	0.9	0.8	2.5	**20.2**
PAD	1.9	1.2	1.5	1.6	**12.4**
Outcome, *n* (col %)					
Falls	177 (3.5)	95 (2.7)	12 (2.3)	44 (6.5)	26 (9.7)
Fractures	197 (3.9)	135 (3.8)	16 (3.0)	29 (4.3)	17 (6.4)

*OSA* obstructive sleep apnoea, *COPD* chronic obstructive pulmonary disease, *PAD* peripheral artery disease, *LCA Class 1* relatively healthy, *LCA Class 2* predominantly respiratory and atopy, *LCA Class 3* predominantly mental health and musculoskeletal, *LCA Class 4* predominantly cardiometabolic

### Individual morbidities

**Fig. 1:**
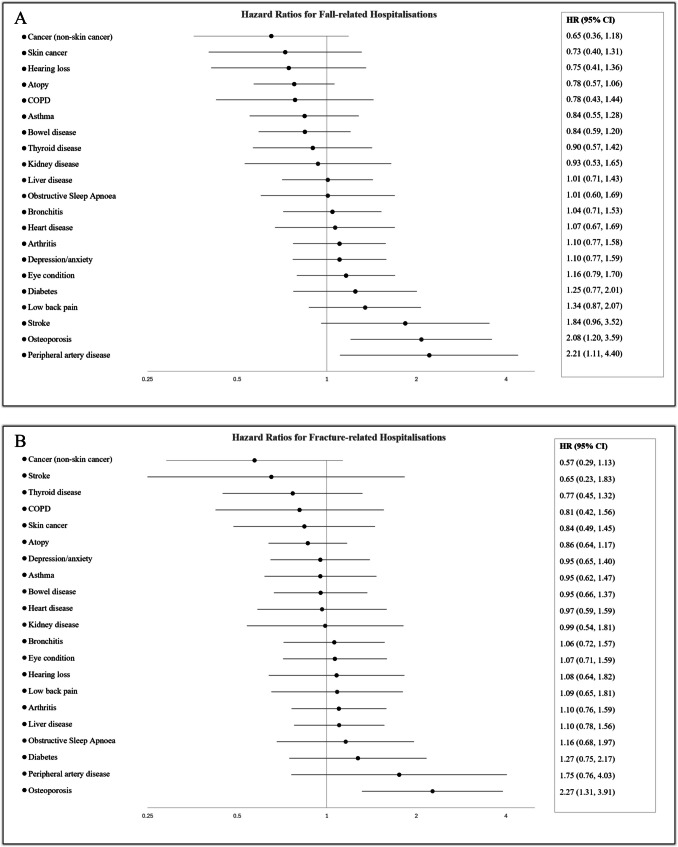
Hazard ratios for **A** fall- and **B** fracture-related hospitalisations for individual conditions, adjusted for multimorbidity count, sex, baseline age, BMI, history of same event 5 years prior to baseline visit, smoking status, physical activity, and alcohol consumption. COPD, Chronic Obstructive Pulmonary Disease

Figure 1 shows the associations between individual morbidities and the risk of (A) fall- and (B) fracture-related hospitalisations, respectively. Associations for individual morbidities were adjusted for total multimorbidity count. For fall-related hospitalisations, the strongest associations were observed for osteoporosis and peripheral artery disease, with hazard ratios of 2.08 (95% CI, 1.20–3.59) and 2.21 (95% CI, 1.11–4.40) respectively. There were also modest associations for stroke, low back pain, and diabetes. The strongest association for an increased risk of fracture-related hospitalisations was for a diagnosis of osteoporosis (HR 2.27 [1.31–3.91]), with modest associations present for peripheral artery disease and diabetes.

### Multimorbidity count

**Fig. 2:**
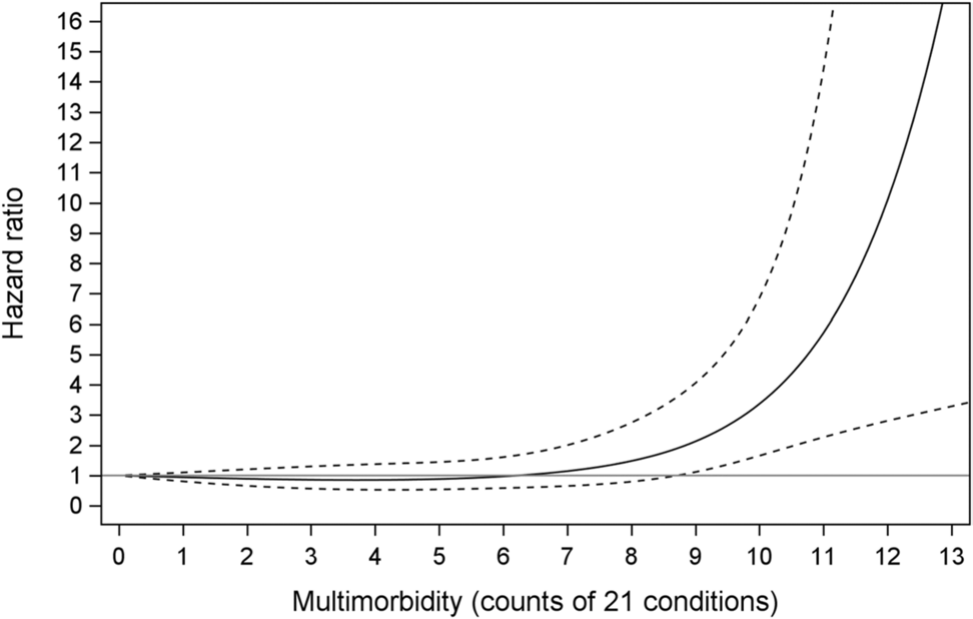
Hazard ratios based on cubic splines to describe the association between multimorbidity count (of 21 conditions) and fracture-related hospitalisations, adjusted for sex, baseline age, BMI, history of same event in 5 years prior to baseline visit, smoking status, physical activity, and alcohol consumption

Regression modelling of the relationship between multimorbidity count and fall-related hospitalisations showed a linear effect. Each one-unit increase in multimorbidity count was associated with a 16% (95% CI, 7.8–25%) increased risk of fall-related hospitalisations (Table 3). In contrast, the association between multimorbidity and fracture-related hospitalisations was non-linear. For up to eight morbidities, there was no evidence of increased fracture risk. For nine or more morbidities, an increasing fracture risk with increasing morbidities was observed; however, only a small number of cases (0.5% of the cohort) had counts at these levels (Fig. 2). Compared with participants with no chronic conditions, having nine morbidities was associated with a hazard ratio of 2.32 (95% CI, 1.22–4.42) for fracture-related hospitalisations.

**Table 3:** Hazard Ratios for fall- and fracture-related hospitalisations according to LCA multimorbidity class and counts (*n* = 4991)

		Model A	Model B	Model C
Fall-related hospitalisations				
LCA MM class	1	Ref	Ref	Ref
	2	0.83 (0.46, 1.51)	0.80 (0.44, 1.45)	0.79 (0.43, 1.44)
	3	2.58 (1.80, 3.68)	1.84 (1.27, 2.67)	1.78 (1.23, 2.59)
	4	3.85 (2.49, 5.94)	2.91 (1.81, 4.68)	2.84 (1.76, 4.59)
MM (count of 21 conditions)	-	1.26 (1.18, 1.34)	1.17 (1.09, 1.26)	1.16 (1.08, 1.25)
Fracture-related hospitalisations				
LCA MM class	1	Ref	Ref	Ref
	2	0.77 (0.46, 1.30)	0.77 (0.46, 1.29)	0.77 (0.46, 1.30)
	3	1.17 (0.78, 1.75)	1.03 (0.68, 1.55)	1.03 (0.68, 1.56)
	4	1.75 (1.06, 2.90)	1.68 (0.98, 2.87)	1.79 (1.04, 3.07)
MM (count of 21 conditions) (non-linear)	Zero conditions	Ref	Ref	Ref
	Three conditions	0.91 (0.61, 1.37)	0.86 (0.57, 1.29)	0.87 (0.57, 1.32)
	Six conditions	1.10 (0.69, 1.77)	0.96 (0.58, 1.59)	0.98 (0.59, 1.63)
	Nine conditions	2.72 (1.52, 4.88)	2.30 (1.21, 4.38)	2.32 (1.22, 4.42)

Values are hazard ratios (95% confidence intervals) obtained using Cox proportional hazards regression. *MM*, multimorbidity; *Ref.*, reference

LCA Class 1: relatively healthy; LCA Class 2: predominantly respiratory and atopy; LCA Class 3: predominantly mental health and musculoskeletal; LCA Class 4: predominantly cardiometabolic

Model A: unadjusted

Model B: adjusted for age, BMI, and sex

Model C: adjusted for sex, age, BMI, history of fall-related hospitalisation 5 years prior to baseline visit, smoking status, physical activity, and alcohol consumption

MM (count of 21 conditions): For falls, hazard ratios represent the change in risk of hospitalisation per each additional morbidity. For fractures, hazard ratios represent the relative risk of hospitalisation for participants when compared to those with 0 morbidities. A number of morbidities were selected to represent increasing disease burden and improve interpretability

### Multimorbidity pattern

**Fig. 3:**
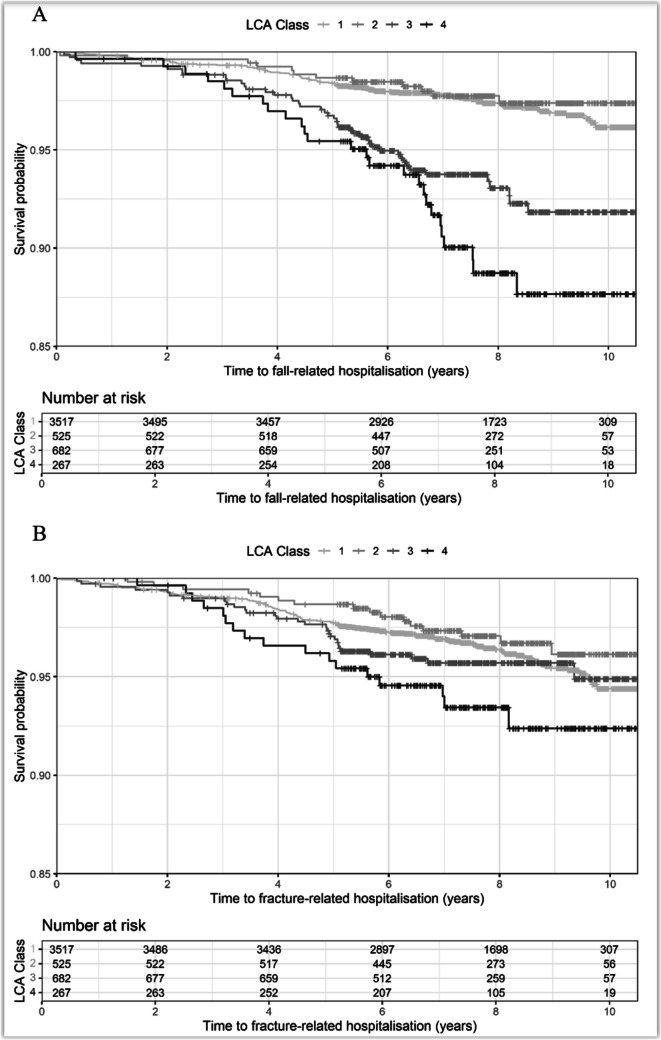
Kaplan–Meier survival curves for the associations between multimorbidity LCA groups with **A** fall- and **B** fracture-related hospitalisations. LCA Class 1: relatively healthy; LCA Class 2: predominantly respiratory and atopy; LCA Class 3: predominantly mental health and musculoskeletal; LCA Class 4: predominantly cardiometabolic

Figure 3 shows Kaplan–Meier survival curves by LCA classes for time to first (A) fall- and (B) fracture-related hospitalisation. There was evidence that participants in LCA class 3 (predominantly mental health and musculoskeletal) and LCA class 4 (predominantly cardiometabolic) were associated with an increased risk of fall-related hospitalisations compared to those in class 1 (relatively healthy). In the fully multivariate-adjusted model (Model C), being in multimorbidity classes 3 and 4 was associated with hazard ratios of 1.78 (95% CI, 1.23–2.59) and 2.84 (95% CI, 1.76–4.59), respectively (Table 2). In addition, there was an association between participants in LCA class 4 and an increased risk of fracture-related hospitalisations (HR 1.79 [1.04–3.07]).

## Discussion

Falls and fractures are associated with increased disability, mortality, and healthcare costs [3]. Investigating the relationship between multimorbidity, falls, and fractures may improve identification of at-risk individuals and lead to earlier interventions. In this prospective study of Australian middle-aged adults, we found that overall multimorbidity count and multimorbidity patterns with a cardiometabolic or mental health and musculoskeletal predominance were associated with an increased risk of fall-related hospitalisations. In addition, the predominantly cardiometabolic multimorbidity class was associated with an increased risk of fractures. Adjusted for the number of comorbidities, most individual morbidities were limited in their association with fall or fracture risk.

Confidence intervals for individual morbidities were often wide, particularly for morbidities with lower prevalence, making it difficult to detect clinically relevant but smaller effect sizes. Despite this, increased risks were observed for osteoporosis and peripheral artery disease. Osteoporosis is recognised as a significant risk factor for falls and fractures [11, 18], as also shown in our study. It is intuitive that the low bone mineral density required for a diagnosis of osteoporosis would lead to a higher risk of fractures, and we postulate that the increased risk of falls is due to concomitant frailty and reduced mobility [34]. Several cardiovascular disorders, including peripheral artery disease, have also been associated with an increased risk of falls [35]. Given the link between peripheral artery disease and other cardiovascular disorders, this risk may be due to syncopal episodes, vascular damage to neural pathways responsible for gait and balance, or medication-related side effects [36].

Stroke [35], low back pain [37], and diabetes [6] have all previously been shown to be associated with an increased risk of falls among community dwelling adults. The modest associations shown in our study support these previous findings. Neurological deficits such as reduced power and proprioception in the lower limbs are common among people with stroke, lead to functional decline, and explain the association between stroke and falls risk. The increased falls risk experienced by diabetics can likely be explained by deficits caused by peripheral neuropathy or hypoglycaemic events caused by inappropriate glycaemic control [38].

Each increase in multimorbidity count was associated with an increased risk of falls, while a multimorbidity count of nine or above was associated with a twofold higher risk of fractures. The cumulative effect of multimorbidity count on the risk of falls has been demonstrated previously [17, 18, 26]. We postulate that this increasing chronic disease burden leads to frailty and the development of functional impairments, which in turn increases the risk of falls and fractures. However, given the variability in effect sizes between individual morbidities on the risk of falls and fractures, multimorbidity count may not always provide the most accurate assessment of risk.

To better differentiate between individuals with similar disease counts but different types of diseases, latent class analysis was used to define four distinct multimorbidity classes. As shown in Table 2, these classes vary both by the mean number of conditions and by the pattern of predominant morbidities. Class 2 (predominantly respiratory and atopy) has a mean of 3.65 morbidities and is characterised by a higher probability of a diagnosis of atopy, asthma, and chronic obstructive pulmonary disease (COPD). Despite evidence to support an association between COPD and an increased risk of falls in elderly individuals [39, 40], when compared to class 1 (relatively healthy), there were no associations between class 2 and an increase in the risk of fall- or fracture-related hospitalisations in the present study. COPD is a progressive disease, primarily of older age, and our findings suggest COPD and other clustered respiratory conditions are not a significant risk factor for falls or fractures in this younger age group.

Participants of class 3 (predominantly musculoskeletal and mental health) and class 4 (predominantly cardiometabolic) had an increased risk of fall-related hospitalisations compared to class 1. These two classes had the highest morbidity burdens of the four classes, and class 4 had a hazard ratio for falls significantly higher than would be expected based on mean multimorbidity count alone. This suggests that multimorbidity classes have a higher discriminative performance in assessing falls risk than generic multimorbidity counts and highlights the importance of cardiometabolic diseases when considering falls risk. Class 3 participants, characterised by higher probabilities of a diagnosis of arthritis, bowel disease, depression/anxiety, eye conditions, and low back pain, would likely have poorer mobility due to chronic pain or sensory impairment due to poor vision, which could explain their higher risk of falls. Class 4 is characterised by higher probabilities of cardiometabolic conditions including liver disease, heart disease, diabetes, obstructive sleep apnoea, and peripheral artery disease. Class 4 had the lowest weekly hours (mean) of moderate/vigorous physical activity reported by the participants of the four classes (7.4 h versus 10.1 h in class 1) [16]. Other factors such as syncope, decreased exercise tolerance, or the reduced peripheral nerve function and balance associated with diabetes are all possible mechanisms to explain the increased falls risk experienced by class 4 participants [41].

A range of mechanisms have been proposed as to how overall multimorbidity increases the risk of falls, including through its relationship with frailty, its interplay with polypharmacy, and its effect on overall physical functioning. Several studies have linked increasing morbidity to a decline in physical functioning using survey data [4, 5]. Components of physical functioning such as mobility, muscle strength, and balance can also be assessed using more objective measures such as the timed up and go test or the five times sit to stand test. Poor performance in these tests has all been shown to be risk factors for falls [42, 43]. As poor physical functioning is likely both a mechanism for how multimorbidity increases the risk of falls and an independent risk factor in its own right, more research is needed to explore the interplay between these factors.

Despite the strong associations between multimorbidity and falls, and contrary to a recently published paper on multimorbidity clusters [29], only participants of class 4 had an increased risk of fracture-related hospitalisations. There are several possible reasons for this. Firstly, our cohort is younger than other studies [22, 28, 29] and therefore has a relatively lower prevalence of osteoporosis (3.4%). We postulate that given the risk for fragility fractures is lower in people with normal bone mineral density, the association between falls and fractures in our cohort is not as strong. Secondly, there exists significant heterogeneity among studies that have derived multimorbidity classes, with the replicability of these analyses questioned by a recent systematic review [27]. Compared to a recent Danish study, our analysis has fewer morbidities included, varies in how the morbidities were defined, and varies in its LCA methodology and chosen multimorbidity classes. Regardless of the variation among studies, it is evident that our predominantly cardiometabolic class 4 has identified individuals with the highest risk of fall- and fracture-related hospitalisations in our cohort.

Our study has several strengths. The inclusion of 21 chronic conditions is more comprehensive than other comparable studies [17, 18, 21, 26] and meets the recommendation of at least 20 by a recent systematic review [27]. In addition, the use of objective measures in the definition of 13 of the 21 conditions gives greater reliability to our multimorbidity analysis. The study is longitudinal and uses standardised coding of hospital admission endpoint captured events several years after baseline assessment, avoiding bias related to self-reporting and loss of follow-up.

However, this study also has several limitations. Our analysis does not consider the severity of the individual morbidities in question or any interventions the patients may have received to reduce their risk of falls and fractures. Our study did not capture emergency department presentations that did not result in an admission, and therefore only falls with relatively serious injury warranting hospitalisation were included. While our overall sample size was relatively large, the number of hospitalisation events was modest, limiting our ability to detect smaller effect sizes or condition-specific risks and contributing to wider confidence intervals in some hazard ratios. Our study was unable to exclude pathological fractures or account for the presence of polypharmacy at the time of hospital admission. We were unable to capture any new morbidities present at hospitalisation that were not present at baseline, and our younger cohort and related low prevalence of osteoporosis reduce the applicability of these results to more elderly individuals. There is also significant heterogeneity in the literature regarding the optimal statistical technique used, methodology, and the number of morbidities included in the determination of multimorbidity profiles [27]. LCA class limits (four in this analysis) may also mean that some conditions that would naturally form their own small clusters are forced into the nearest statistically comparable group.

In summary, in this middle-age cohort, the number of co-morbidities as well as certain multimorbidity patterns was associated with higher risk for fall- and fracture-related hospitalisations. Recently published global guidelines for falls prevention and management recommend regular risk stratification of older adults opportunistically and at annual health visits in the primary care setting through questions on falls history and fear of falling [44]. Our results suggest that multimorbidity count and pattern should also be considered when assessing a patient’s risk of falls and fractures. Further research is needed to assess the mechanisms through which this risk is conferred, including through the interplay with overall physical functioning.

## Data Availability

The data that supports the findings of this study was used under license, and so is not publicly available. The data is, however, available from the authors upon reasonable request, with permission from the Busselton Population Medical Research Institute.
